# Ledderhose's Disease

**DOI:** 10.7759/cureus.64593

**Published:** 2024-07-15

**Authors:** Manare Jaai, Khaoula Kassi, Araj Aymane, Ahmed Amine El Oumri

**Affiliations:** 1 Physical Medicine and Rehabilitation, Faculty of Medicine of Oujda/Mohammed VI University Hospital of Oujda/Mohammed First University of Oujda, Oujda, MAR

**Keywords:** ultrasonography, plantar fibromatosis, swelling, foot mass, heel pain

## Abstract

Ledderhose disease, also known as plantar fibromatosis, is a rare fibroproliferative disorder characterized by the development of fibrous nodules within the plantar fascia of the foot. These nodules cause discomfort, pain, and impaired mobility, particularly during activities like walking, and are often associated with other fibromatoses, such as Dupuytren's disease.

In this case, a 60-year-old woman presented with significant plantar pain exacerbated by walking, along with swelling in the arch of her foot. The diagnosis involved a clinical examination that revealed nodules and tenderness in the plantar fascia, and ultrasound imaging confirmed the presence of fibrotic tissue.

Due to the patient's preference for non-surgical management, a conservative approach was adopted. This included the use of medications, orthotic devices, and physical therapy.

This case underscores the effectiveness of non-surgical interventions in managing Ledderhose disease, highlighting the importance of personalized treatment plans tailored to patient preferences.

## Introduction

Ledderhose disease is an uncommon, benign, and hyperproliferative disorder affecting plantar aponeurosis, characterized by abnormal cellular proliferation [1,2]. First described by the German physician Georg Ledderhose in 1897 [3]. The condition typically exhibits a male predominance and affects individuals aged between 30 and 50 [1]. It involves fibroblastic proliferation of type 3 collagen with intense neovascularization [3].

Diagnosis relies on clinical evaluation, notably the palpation of firm nodular masses within the plantar fascia, typically located in the medial and central regions of the foot [2]. Radiographs may support a diagnosis. Ultrasound imaging typically reveals nodular masses within the plantar fascia and assesses changes in adjacent structures and the surrounding soft tissues [3]. While MRI is excellent for showing deeper extensions [4], histological examination of the nodule can confirm the presence of fibrosis [5].

We report a case of Ledderhose disease in a 60-year-old patient, emphasizing the diagnostic complexities and the effectiveness of non-surgical treatment approaches.

## Case presentation

A 60-year-old female patient with a medical history significant for diabetes, hypertension, medial and paramedian disc herniation at the L4-L5 level, and a narrow lumbar canal presented with a two-year history of progressively worsening plantar pain on the medial aspect of the left foot, hindering walking and relieved notably with rest.

**Figure 1:**
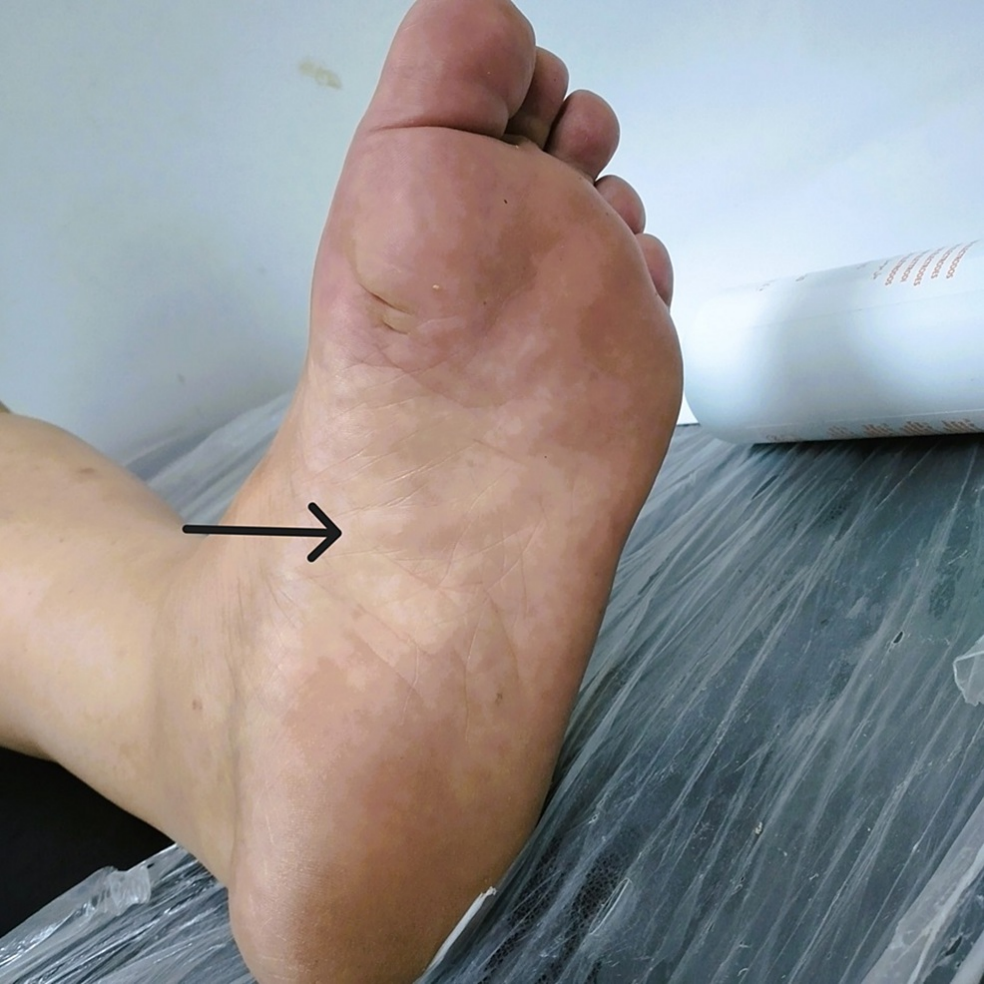
Close-up view of the medial aspect of the left foot showing subcutaneous nodules and retraction

On clinical examination, vital signs were within the normal range, but the patient exhibited signs of obesity; the body mass index was 32. Thickening of the plantar fascia and a tender subcutaneous nodule on the medial aspect of the left foot (Figure 1) were noted upon palpation. Range of motion testing revealed no notable restrictions in foot and toe movement. Sensory and motor functions of the foot and lower extremities were intact without neurological deficits. Vascular perfusion was normal. An altered gait favoring the affected foot with a slight limp was observed during walking. The physical examination of the other limbs was unremarkable.

Laboratory tests, including complete blood count (CBC), erythrocyte sedimentation rate (ESR), and C-reactive protein (CRP), yielded results within normal limits.

**Figure 2:**
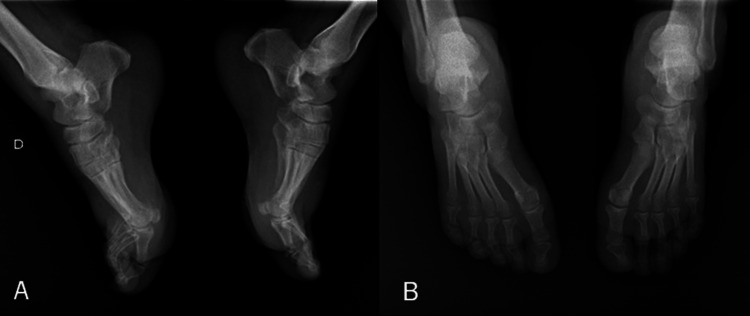
Anteroposterior and lateral radiographs of the left and right without abnormalities (A) Posteroanterior X-ray; (B) Lateral X-ray

X-ray examination of the foot (Figure 2) showed no bone deformities.

**Figure 3:**
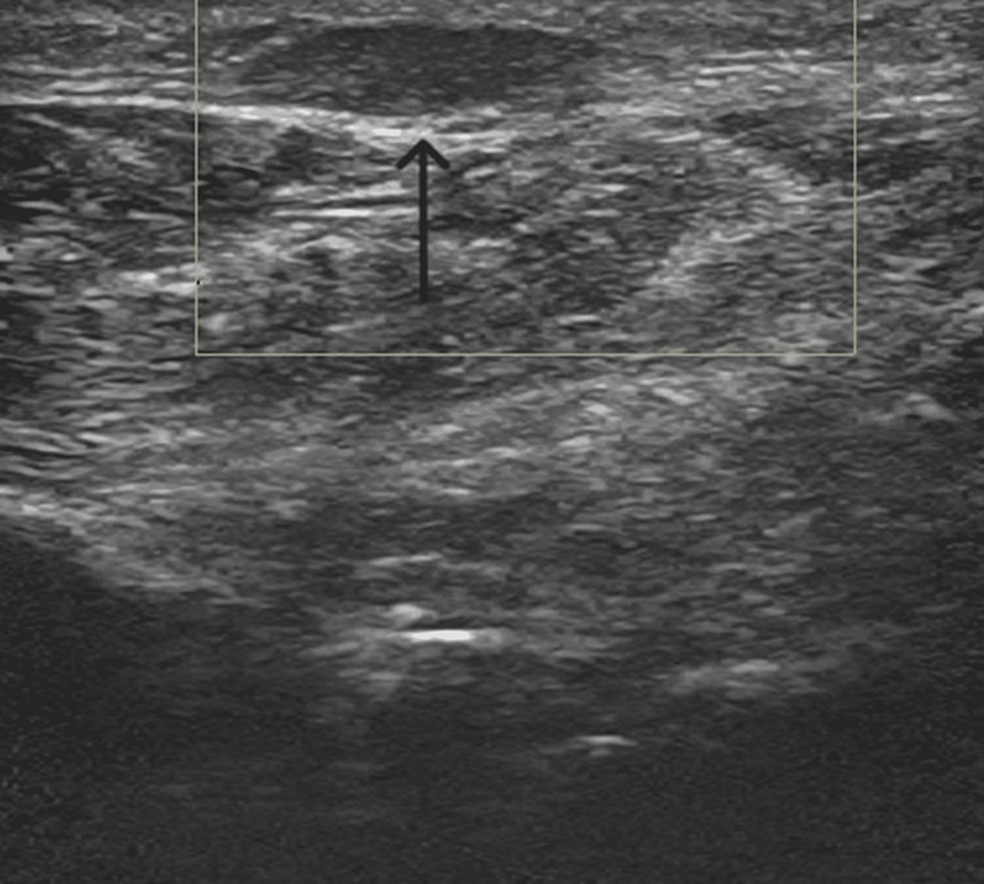
An ultrasound of the arch of the left foot was performed and found a hypoechoic and homogeneous nodule within the plantar fascia. Color and power Doppler imaging revealed no internal blood flow.

**Figure 4:**
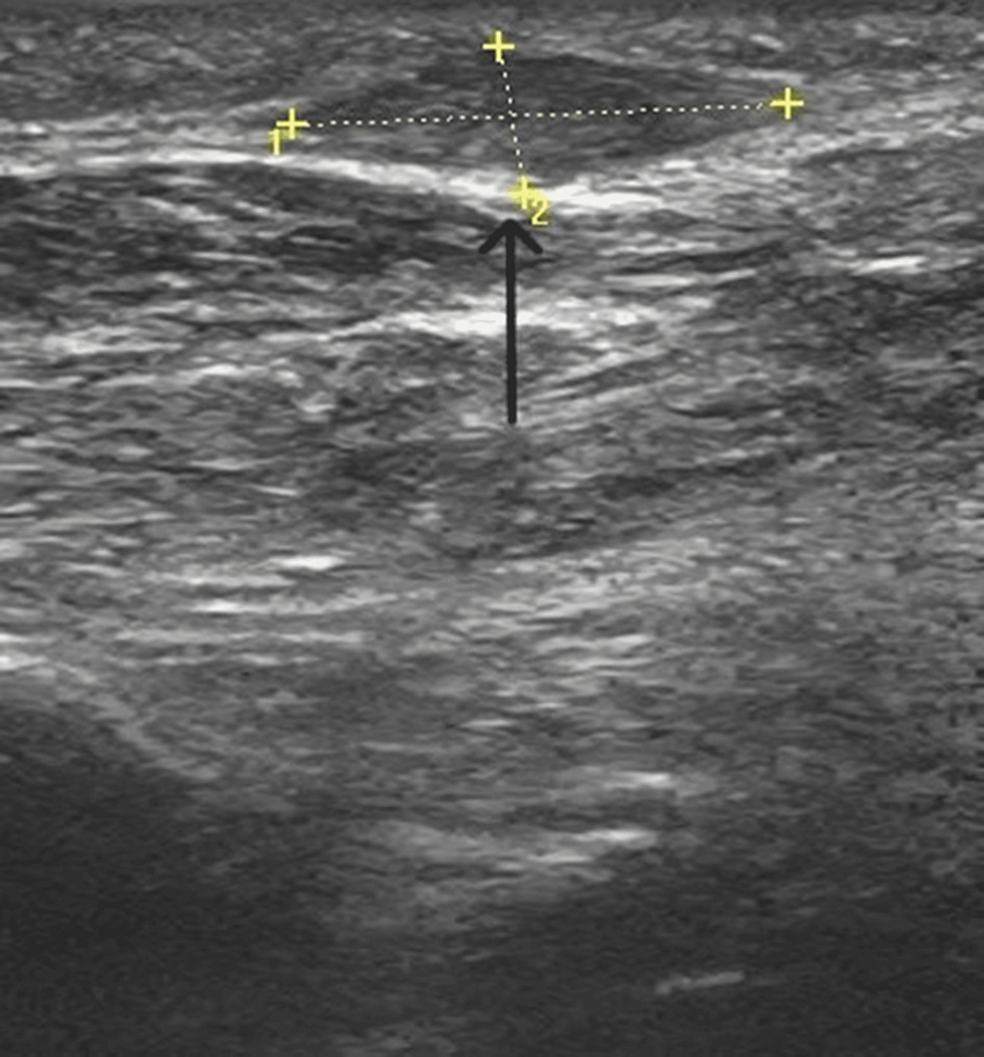
Ultrasound of the left foot revealed a nodule measuring approximately 1.40 cm in its longest axis and 0.50 cm in its shortest axis.

Ultrasound imaging of the arch (Figures 3, 4) revealed a hypoechoic, homogeneous nodule within the plantar fascia with no internal blood flow on Doppler imaging. The nodule measured approximately 1.40 cm in length and 0.50 cm in width.

In our patient's management, we initiated treatment with NSAIDs (ibuprofen 400 mg twice daily) along with paracetamol-codeine (400 mg | 20 mg) twice daily for two weeks. Additionally, anti-shock foot orthoses were prescribed. A regimen of physical therapy was integrated into the treatment plan to alleviate symptoms and enhance gait.

The patient underwent 20 sessions of physical therapy, lasting 30 to 60 minutes each, scheduled 2 to 3 times weekly. Treatment goals included improving range of motion, strengthening intrinsic and extrinsic foot muscles, stretching the plantar fascia, and optimizing gait mechanics.

After one month of evaluation, the patient reported reduced symptoms, including decreased foot discomfort and improved mobility during daily activities. Physical examination revealed diminished tenderness upon palpation of the plantar fascia. Gait analysis indicated a more normalized stride with reduced limping and improved weight-bearing on the affected foot.

## Discussion

Ledderhose disease is a rare, benign, hyperproliferative disorder of the plantar aponeurosis [1-2]. First described by the German physician Georg Ledderhose in 1897 [1], it primarily affects middle-aged individuals (typically between 30 and 50 years old), with a higher incidence observed among males [1]. Bilateral involvement occurs in approximately 25% of patients [1-2].

Although the etiopathology of Ledderhose disease remains uncertain [1], genetic predisposition and alterations in the collagen profile of the plantar aponeurosis are hypothesized as causal factors [3]. Increased production of certain cell growth factors is believed to contribute to fibromatosis formation and progressive contracture development [1]. Ledderhose disease is frequently associated with conditions such as alcoholism, frozen shoulder, Dupuytren's disease, epilepsy, diabetes mellitus, and Peyronie's disease [2].

This condition manifests as gradual nodules developing in the inner part of the plantar fascia, potentially resulting in its thickening. Fibromatosis induces toe contractures and presents symptoms such as foot pain and swelling, sometimes making walking difficult. Early-stage indicators involve localized pressure and swelling, while the advanced stage is marked by nodule formation and plantar aponeurosis contracture [1].

Plantar fibromatosis typically exhibits a characteristic ultrasound pattern, serving as a quick and non-invasive method to confirm the clinical diagnosis. Reed provided the initial ultrasound depiction [3]. The nodule usually appears as a single hypoechoic mass, measuring approximately one centimeter in diameter. It exhibits an internal structure of varied density with a few thin hyperechoic partitions. The fibrous growth of the nodule adheres along the length of the plantar aponeurosis and features well-defined borders. Typically, there is no evidence of calcification or fluid accumulation within the nodule. Color and power Doppler imaging typically reveal no internal blood flow [2].

MRI excels at revealing deep extensions, which are characteristic of advanced and aggressive types of plantar fibromatosis. However, due to its availability and cost-effectiveness, ultrasound remains the preferred imaging modality for most patients. On MRI, plantar fibromatosis typically appears as a distinct nodule closely associated with the plantar fascia. It exhibits low signal intensity on T1-weighted sequences and ranges from low to intermediate signal intensity on T2-weighted sequences [4].

Histological examinations of fibromatosis reveal a proliferation of myofibroblasts characterized by elongated oval nuclei and an abundance of type III collagen. Atypical mitotic figures, irregular nuclear morphology, and poorly defined spindle-shaped fibroblast bundles suggest a higher degree of cellular differentiation, aiding in differentiation from fibrosarcoma. Dr. Zgonis led a comprehensive investigation [5], incorporating histochemical, immunohistochemical, and ultrastructural analyses, yielding results notably similar to those observed in Dupuytren's disease [2,3].

In the initial stages of Ledderhose disease, conservative treatment strategies are typically favored as they aim to manage symptoms and slow disease progression. However, it's crucial to recognize that nodules may grow or relapse even without surgery [6].

These approaches encompass various methods and techniques, including the administration of non-steroidal anti-inflammatory drugs (NSAIDs), such as ibuprofen, and topical agents [7].

Orthotic support and cutouts reduce fascial tension, providing pain relief, yet they do not completely halt the progression of the lesion [6].

The rehabilitation program aims to achieve pain relief, manage contractures, strengthen muscles, improve functionality and mobility, and correct gait. It includes analgesic physiotherapy such as transcutaneous electrical nerve stimulation (TENS), which alleviates pain by activating neural pathways to reduce heightened pain sensitivity [8]. Deep oscillation therapy stimulates tissue movements effectively, potentially enhancing local blood flow in the skin, muscles, and blood vessels [9]. Additionally, the program incorporates exercises to strengthen leg stabilizers, improve leg flexibility, stretch the Achilles tendon, and correct gait. Activities also include bicycle routines, pedal exercises, toe curls, standing stretches, calf stretches, plantar fascia massage, and supervised ambulation to enhance overall well-being [7]. Corticosteroid injections guided by ultrasound alter the production of growth factors and cytokines, thereby reducing symptoms and lesion size. However, relapse remains possible, with studies showing a 50% decrease in lesion recurrence within the first three years [10]. These injections may also be beneficial in lowering systemic inflammation levels among patients with chronic conditions [11].

In 2012, Knobloch and Vogt conducted a study showcasing the advantages of extracorporeal shock wave therapy (ESWT) in alleviating pain and softening lesions associated with plantar fibromatosis [12]. As with other conditions, the use of systemic analgesics during therapy sessions is recommended [13].

Surgical interventions become necessary when conservative treatments prove ineffective and pain persists. There are three main surgical procedures: local excision, wide excision, and complete fasciectomy. Several studies have indicated that local nodule excision carries the highest recurrence rates, ranging from 57% to 100% [14,15].

The wide excision procedure entails removing the nodule along with a 2-3 cm margin of surrounding tissue. Studies have indicated lower recurrence rates compared to local excision, spanning from 8% to 80% [16,17].

A complete fasciectomy stands as the most invasive. According to Aluiso et al., it boasts the lowest recurrence rates, ranging approximately from 0% to 50% [15]. Endoscopic plantar fasciectomy offers smaller wounds, better cosmetics, and reduces scar-related complications. However, it's not suitable for lesions invading muscle, skin, or nerves and requires skilled arthroscopists due to its technical demands [17].

## Conclusions

Plantar fibromatosis is a benign lesion of unknown origin. The diagnosis of this disease is based on a clinical examination. Radiographs are not necessary to make the diagnosis, but they may be indicated to exclude bone disease. Ultrasound and magnetic resonance imaging (MRI) can be helpful in confirming the diagnosis. Biopsies may be performed to rule out malignant tumors. The treatment approach should be guided by the functional impact, ranging from abstention to surgical excision.
